# *PARP1* gene polymorphisms and neuroblastoma susceptibility in Chinese children

**DOI:** 10.7150/jca.34222

**Published:** 2019-07-10

**Authors:** Jiwen Cheng, Zhenjian Zhuo, Pu Zhao, Jinhong Zhu, Yijuan Xin, Jiao Zhang, Peng Li, Ya Gao, Jing He, Baijun Zheng

**Affiliations:** 1 Department of Pediatric Surgery, the Second Affiliated Hospital of Xi'an Jiaotong University, Xi'an 710004, Shaanxi, China; 2 Department of Pediatric Surgery, Guangzhou Institute of Pediatrics, Guangdong Provincial Key Laboratory of Research in Structural Birth Defect Disease, Guangzhou Women and Children's Medical Center, Guangzhou Medical University, Guangzhou 510623, Guangdong, China; 3 Department of Neonatology, Shaanxi Provincial People's Hospital, Xi'an 710068, Shaanxi, China; 4 Department of Clinical Laboratory, Molecular Epidemiology Laboratory, Harbin Medical University Cancer Hospital, Harbin 150040, Heilongjiang, China; 5 Clinical Laboratory Medicine Center of PLA, Xijing Hospital, Air Force Medical University, Xi'an 710032, Shaanxi, China; 6 Department of Pediatric Surgery, the First Affiliated Hospital of Zhengzhou University, Zhengzhou 450052, Henan, China

**Keywords:** *PARP1*, polymorphism, neuroblastoma, susceptibility, DNA repair

## Abstract

Neuroblastoma is a heterogeneous cancer frequently occurring in childhood. Germline mutations of *PARP1* oncogene are implicated in several types of cancer. However, whether common single nucleotide polymorphisms (SNPs) in *PARP1* gene are associated with neuroblastoma risk has received relatively few attentions. In this multi-center study, we aimed to elucidate the contributing role of *PARP1* SNPs in neuroblastoma risk. We successfully genotyped three potentially functional *PARP1* SNPs (rs1136410 A>G, rs2666428 T>C, rs8679 A>G) in 469 neuroblastoma cases and 998 controls. We did not detect any significant association between the analyzed SNPs and neuroblastoma risk in single SNP analysis. However, stratified analysis revealed that rs1136410 AG/GG carriers were more likely to develop tumors arising from mediastinum (AG/GG vs. AA: adjusted OR=1.65, 95% CI=1.06-2.56, *P*=0.028). Moreover, rs2666428 TC/CC carriers were at significantly lower risk to develop tumors from “other sites” (TC/CC vs. TT: adjusted OR=0.44, 95% CI=0.20-0.96, *P*=0.040). Our findings failed to provide evidence of the conferring role of the *PARP1* gene polymorphisms in the risk of neuroblastoma. Further investigations of the association between *PARP1* gene SNPs and neuroblastoma risk are warranted.

## Introduction

Neuroblastoma, arising from neural crest progenitor cells, is one of the most common extracranial solid tumors 1, 2. Neuroblastoma mainly affects children under age of five years. Neuroblastoma accounts for about 8-10% of childhood malignancies and is related to 12-15% of cancer related childhood mortality 3-5. The clinical patterns of neuroblastoma vary widely, ranging from spontaneous regression to relapse and therapy-resistant disease 6. Neuroblastoma is classified into low-, intermediate-, and high-risk group 7, and high-risk neuroblastoma constitutes approximately 50% of cases 8. Despite intensive and multi-modal therapy, 5-year survival rates of high-risk neuroblastoma patients were less than 40% 9. One major characteristic of these high-risk neuroblastomas are their metastases to bone, noncontiguous lymph nodes, and bone marrow 2.

Neuroblastoma is a complex disease resulting from the interactions of environmental and genetic risk factors. Environmental factors such as maternal medication, childhood infections, and poor living habitat might increase the risk of developing neuroblastoma 10, 11. However, only a small portion of the exposed children finally develop neuroblastoma, suggesting the involvement of other risk factors. Growing evidence indicates that genetic factors also play an important role in the initiation and development of neuroblastoma 12, 13. For instance, *PHOX2B* and *ALK* gene mutations contributed to tumorigenesis of neuroblastoma in a fraction of patients 14-16. Moreover, candidate gene approaches also identified polymorphisms in *NEFL* 17 and *CDKN1B* 18 genes significantly associated with neuroblastoma susceptibility. More analysis methods, such as fine mapping, also identified neuroblastoma-associated functional risk SNPs in *BARD1* 19.

DNA repair pathways are critical mechanisms for maintaining genome integrity 20. Of DNA repair pathways, base excision repair (BER) is the major repair mechanism to repair DNA damage caused by ionizing radiation or chemical alterations of a single base 21, 22. The human poly(ADP-ribose) polymerase-1 (PARP1) is a key protein in BER system, recognizing DNA double strand break 23. The activation of PARP1 is one of the early responses to DNA damage 24. PARP1 catalyzes poly(ADPribosyl)ation, a quick DNA-damage dependent posttranslational self modification, as well as modification of histones and other nuclear proteins. PARP1 protein is essential in mediating several critical cellular mechanisms, including DNA damage recognition, DNA damage repair, mitotic apparatus function and cell death pathways 25, 26. A number of single nucleotide polymorphisms (SNPs) in the *PARP1* gene are reported to be associated with the risk of several types of cancer 27. However, till now, no reports have evaluated the association of *PARP1* gene polymorphisms with neuroblastoma risk. Thus, we performed a multi-center case-control study to explore whether *PARP1* gene polymorphisms could predispose to neuroblastoma risk.

## Materials and Methods

### Study populations

In total, 469 patients with neuroblastoma were enrolled in three independent medical centers in China from September 2009 to March 2018, without receiving prior therapy. The three participating centers are as follows: Guangzhou Women and Children's Medical Center, Guangdong province (275 cases and 531 controls) 28-30; The First Affiliated Hospital of Zhengzhou University, Henan province (118 cases and 281 controls) 31; Second Affiliated Hospital of Xi'an Jiao Tong University, Shaanxi province (76 cases and 186 controls) 32. During the same period, 998 controls were recruited from the three same geographical regions, respectively, and frequency-matched to the cases according to age (±5 years) and gender. After providing signed informed consent, all the participants' parents were personally interviewed to collect clinical information. Blood was drawn from each participant for DNA extraction and genotyping. Details of the selection criteria have been described previously 33-35. This study was approved by the Institutional Review Board of the three participating hospitals.

### SNP selection

SNPs with a minor allele frequency (MAF) > 5% of Chinese Han in the *PARP1* gene were selected from NCBI dbSNP database. An online software SNPinfo (https://snpinfo.niehs.nih.gov/) was adopted to predict putative functional potential of SNPs 36. In final, three potentially functional SNPs (rs1136410 A>G, rs2666428 T>C, rs8679 A>G) in the *PARP1* gene were chosen for analysis. The rs1136410 A>G might affect splicing activity, rs2666428 T>C and rs8679 A>G might affect the microRNA binding site activity. There is no significant linkage disequilibrium (R^2^<0.8) among these three selected polymorphisms in the *PARP1* gene (R^2^=0.051 between rs8679 and rs1136410; R^2^=0.26 between rs8679 and rs2666428; R^2^=0.196 between rs1136410 and rs2666428) (**Supplemental Figure 1**).

### Genotyping

Genomic DNA derivation from EDTA-peripheral blood was conducted using TIANamp Blood DNA Kit (TianGen Biotech Co. Ltd., Beijing, China). The SNPs were genotyped using TaqMan platform (Applied Biosystems) with a 384-well plate 37-40. Investigators were blind to case-control status of the study samples. For the purpose of quality control, eight water-only negative controls were used in each plate. About 10% of samples were selected randomly to be genotyped a second time, and the concordance rate was 100%.

### Statistical analysis

The deviation of the SNP genotypes from the Hardy-Weinberg equilibrium was assessed using the goodness-of-fit χ^2^ test in controls. Two-sided chi-square test was conducted to compare the distribution of demographic variables and allele frequencies between the two groups. Odds ratios (ORs), 95% confidence intervals (CIs) and *P* values were calculated. Testing for association between SNPs and neuroblastoma risk was performed using unconditional logistic regression analysis with adjustment for age and gender. Stratified analyses were also conducted by age, gender, tumor sites, and clinical stages. Analyses were carried out using the version 9.4 SAS software (SAS Institute, Cary, NC). The significant threshold was *P*< 0.05 (two-sided).

## Results

### Correlation of *PARP1* gene polymorphisms with neuroblastoma susceptibility

The details of the demographic characteristics for combined subjects and Shaanxi subjects are summarized in **Supplemental Table 1**, while information for Guangdong and Henan subjects were described in our former publication 29-31. **Table 1** presents the genotype frequencies of *PARP1* gene polymorphisms in cases and controls and their association with neuroblastoma susceptibility. The genotype frequency distribution for all SNPs were conformed to Hardy-Weinberg equilibrium (rs1136410, *P*=0.669; rs2666428, *P*=0.569; rs8679, *P*=0.256). Our results indicated that participants harboring the three *PARP1* SNPs were not associated with risk of neuroblastoma in Chinese population, either in single or combined genotype analysis.

### Stratification analysis

We further investigated the effects of three *PARP1* SNPs on the neuroblastoma risk after stratified by age, gender, tumor sites, and INSS stages (Table 2). We detected that the rs1136410 AG/GG genotypes were significantly associated with an increased risk of a tumor originating in the mediastinum (AG/GG vs. AA: adjusted OR=1.65, 95% CI=1.06-2.56, *P*=0.028). As to the rs2666428, negative association was detected in tumor that develops from others (TC/CC vs. TT: adjusted OR=0.44, 95% CI=0.20-0.96, *P*=0.040). No significant association was observed in the stratified analysis of rs8679 and combined risk genotypes.

## Discussion

In the current study, we comprehensively evaluated the association between the genetic variants in *PARP1* gene and neuroblastoma risk. Our data reveal no significant association between the SNPs in *PARP1* gene and the risk of neuroblastoma. To our knowledge this is the first evaluation of *PARP1* SNPs in relation to neuroblastoma risk.

The human *PARP1* gene, also known as* ADPRT*, is located at chromosome 1q41-42 41. It spans 47.3kb and consists of 23 exons. Its encoding protein, PARP1, consists of three domains: an N-terminal DNA binding domain, a central auto-modification domain and a C-terminal catalytic domain 42, 43.

To date, there are at least 439 SNPs found in the *PARP1* gene, including 17 non-synonymous SNPs (http://www.ncbi.nlm.nih.gov/SNP). Among them, Val762Ala (rs1136410) polymorphism was one of the most investigated SNP. *PARP1* Val762Ala leads to a T-to-C transition at codon 762 that causes valine to alanine amino acid substitution. Such substitution was reported to be associated with an altered activity of PARP1 protein 44, 45. By now, several case-control studies have been performed to investigate the role of *PARP1* gene Val762Ala in cancer risk 27. Hao et al. 46 found that *PARP1* Val762Ala was associated with an increased risk of esophageal squamous cell carcinoma in a case-control study of 419 patients and 480 healthy controls. In a study conducted in the USA, Lockett et al. 47 found that *PARP1* Val762Ala genetic variant contributes to prostate cancer susceptibility and alters ADPRT/PARP-1 enzyme function in response to oxidative damage. In a study including 99 breast cancer cases and 96 healthy controls from Saudi population, Mohammad Alanazi et al. 48 reported for the first time that the *PARP1* Ala762Ala genotype significantly contributes to breast cancer susceptibility.

Herein, we for the first time investigated whether *PARP1* gene SNPs (rs1136410, rs2666428, rs8679) could affect the neuroblastoma susceptibility in Chinese children. Our results indicated no association between these three *PARP1* gene SNPs and neuroblastoma risk. Several reasons might help to explain this null finding. First, the relatively small sample size might make it difficult to detect the weak association. Second, other risk factors not analyzed in the present study may be required for *PARP1* gene SNPs to exert a significant effect. In a study using a bladder cancer case-control series (752 cases and 704 controls) as well as a breast cancer case-control series (257 cases and 512 controls) from UK, Mark et al. 49 found that *PARP1* rs8679 was associated with increased bladder cancer and breast cancer risk. The conflicting role of rs8679 in this current study and the study conducted in UK may be ascribed to the ethnicity difference. Intriguingly, stratified analysis indicated that individuals harboring the rs1136410 AG/GG alleles were more likely to have tumor that develops from mediastinum. In the stratified analysis of rs2666428, we found a decreased association with tumor develops from others. Such conflicting role might also be the small sample size in the stratified analysis.

The study had several drawbacks. The main limitation was the relatively small sample size, which impairs our capacity to detect weak association between *PARP1* gene SNPs and neuroblastoma risk. Additionally, with the available data, we could only investigate the genetic information. It is far more enough to elucidate the etiology of neuroblastoma as neuroblastoma is a heterogeneous disease caused by complex between genes or gene-environment interactions. Another limitation is that only three SNPs in *PARP1* gene were analyzed. More potentially functional SNPs in *PARP1* gene are needed to be investigated. Finally, we only included Chinese children in this study. Therefore, the conclusions obtained here should be interpreted with cautious when extrapolated to other populations.

In all, this study suggests that *PARP1* polymorphisms may be weakly associated with neuroblastoma risk. Future studies are needed to validate our findings and target the functional role of *PARP1* polymorphisms in neuroblastoma risk.

## Supplementary Material

Supplementary figure and table.

## Figures and Tables

**Table 1:** Association between *PARP1* gene polymorphisms and neuroblastoma risk

Genotype	Cases (N=469)	Controls (N=998)	*P*^ a^	Crude OR (95% CI)	*P*	Adjusted OR (95% CI) ^b^	*P*^ b^
rs1136410 (HWE=0.669)							
AA	136 (29.00)	330 (33.07)		1.00		1.00	
AG	244 (52.03)	482 (48.30)		1.23 (0.96-1.58)	0.110	1.23 (0.95-1.58)	0.116
GG	89 (18.98)	186 (18.64)		1.16 (0.84-1.60)	0.363	1.17 (0.84-1.61)	0.353
Additive			0.276	1.09 (0.94-1.28)	0.260	1.10 (0.94-1.28)	0.255
Dominant	333 (71.00)	668 (66.93)	0.119	1.21 (0.95-1.54)	0.119	1.21 (0.95-1.53)	0.122
Recessive	380 (81.02)	812 (81.36)	0.877	1.02 (0.77-1.35)	0.876	1.03 (0.78-1.36)	0.846
rs2666428 (HWE=0.569)							
TT	305 (65.03)	636 (63.73)		1.00		1.00	
TC	149 (31.77)	325 (32.57)		0.96 (0.75-1.21)	0.710	0.96 (0.75-1.21)	0.712
CC	15 (3.20)	37 (3.71)		0.85 (0.46-1.56)	0.593	0.85 (0.46-1.57)	0.600
Additive			0.827	0.94 (0.77-1.15)	0.561	0.94 (0.77-1.15)	0.566
Dominant	164 (34.97)	362 (36.27)	0.627	0.95 (0.75-1.19)	0.628	0.95 (0.75-1.19)	0.631
Recessive	454 (96.80)	961 (96.29)	0.623	0.86 (0.47-1.58)	0.623	0.86 (0.47-1.59)	0.630
rs8679 (HWE=0.256)							
AA	407 (86.78)	871 (87.27)		1.00		1.00	
AG	60 (12.79)	125 (12.53)		1.03 (0.74-1.43)	0.873	1.03 (0.74-1.43)	0.872
GG	2 (0.43)	2 (0.20)		2.14 (0.30-15.25)	0.448	2.18 (0.31-15.55)	0.437
Additive			0.732	1.06 (0.78-1.45)	0.710	1.06 (0.78-1.46)	0.705
Dominant	62 (13.22)	127 (12.73)	0.792	1.05 (0.76-1.45)	0.791	1.05 (0.75-1.45)	0.789
Recessive	467 (99.57)	996 (99.80)	0.439	2.13 (0.30-15.19)	0.450	2.17 (0.31-15.49)	0.438
Combined effect of risk genotypes ^c^							
0-1	115 (24.52)	281 (28.16)		1.00		1.00	
2-3	354 (75.48)	717 (71.84)	0.144	1.21 (0.94-1.55)	0.144	1.21 (0.94-1.55)	0.148

OR, odds ratio; CI, confidence interval; HWE, Hardy-Weinberg equilibrium. ^a^ χ^2^ test for genotype distributions between neuroblastoma patients and cancer-free controls. ^b^ Adjusted for age and gender. ^c^ Risk genotypes were rs1136410 AG/GG, rs2666428 TC/TT and rs8679 AG/GG.

**Table 2:** Stratification analysis for association between *PARP1* gene genotypes and neuroblastoma susceptibility

Variables	rs1136410 (case/control)		AOR (95% CI)^a^	*P*^a^	rs2666428 (case/control)		AOR (95% CI)^a^	*P*^a^	rs8679 (case/control)		AOR (95% CI)^a^	*P*^a^	Risk genotypes (case/control)		AOR (95% CI)^a^	*P*^a^
	AA	AG/GG			TT	TC/CC			AA	AG/GG			0-1	2-3		
Age, month																
≤18	53/128	116/262	1.06 (0.72-1.57)	0.757	109/244	60/146	0.92 (0.63-1.34)	0.650	141/348	28/42	1.64 (0.98-2.75)	0.061	43/114	126/276	1.20 (0.80-1.81)	0.381
>18	83/202	217/406	1.30 (0.96-1.77)	0.087	196/392	104/216	0.97 (0.72-1.29)	0.819	266/523	34/85	0.80 (0.52-1.23)	0.310	72/167	228/441	1.21 (0.88-1.66)	0.252
Gender																
Female	62/138	134/276	1.08 (0.75-1.55)	0.681	126/272	70/142	1.07 (0.75-1.52)	0.722	175/372	21/42	1.07 (0.61-1.86)	0.816	56/119	140/295	1.01 (0.69-1.47)	0.968
Male	74/192	199/392	1.32 (0.96-1.81)	0.091	179/364	94/220	0.87 (0.64-1.17)	0.343	232/499	41/85	1.03 (0.69-1.54)	0.896	59/162	214/422	1.39 (0.99-1.95)	0.060
Sites of origin																
Adrenal gland	52/330	110/668	1.03 (0.72-1.48)	0.857	100/636	62/362	1.10 (0.78-1.56)	0.571	144/871	18/127	0.86 (0.51-1.46)	0.579	45/281	117/717	1.01 (0.69-1.46)	0.972
Retroperitoneal	40/330	98/668	1.21 (0.82-1.78)	0.351	89/636	49/362	0.95 (0.66-1.38)	0.801	118/871	20/127	1.14 (0.68-1.90)	0.614	32/281	106/717	1.30 (0.85-1.97)	0.227
Mediastinum	28/330	93/668	**1.65 (1.06-2.56)**	**0.028**	78/636	43/362	0.98 (0.66-1.45)	0.905	102/871	19/127	1.32 (0.78-2.24)	0.296	24/281	97/717	1.60 (1.00-2.55)	0.050
Others	13/330	27/668	1.03 (0.53-2.02)	0.931	32/636	8/362	**0.44 (0.20-0.96)**	**0.040**	37/871	3/127	0.56 (0.17-1.85)	0.343	12/281	28/717	0.92 (0.46-1.84)	0.823
Clinical stage																
I+II+4s	68/330	165/668	1.21(0.88-1.65)	0.241	150/636	83/362	0.97 (0.72-1.31)	0.833	202/871	31/127	1.07 (0.70-1.63)	0.758	57/281	176/717	1.22 (0.88-1.70)	0.231
III+IV	62/330	154/668	1.21 (0.88-1.68)	0.243	144/636	72/362	0.88 (0.64-1.20)	0.402	190/871	26/127	0.92 (0.59-1.45)	0.726	55/281	161/717	1.13 (0.81-1.58)	0.484

AOR, adjusted odds ratio; CI, confidence interval. ^a^ Adjusted for age and gender, omitting the corresponding stratify factor.

## Abbreviations

BER: base excision repair
*PARP1*: poly(ADP-ribose) polymerase-1
SNP: single nucleotide polymorphism
MAF: minor allele frequency
OR: odds ratio
CI: confidence interval
